# Label-Free and Sensitive Determination of Cadmium Ions Using a Ti-Modified Co_3_O_4_-Based Electrochemical Aptasensor

**DOI:** 10.3390/bios10120195

**Published:** 2020-11-30

**Authors:** Yang Liu, Dongwei Zhang, Jina Ding, Kashif Hayat, Xijia Yang, Xuejia Zhan, Dan Zhang, Yitong Lu, Pei Zhou

**Affiliations:** 1 School of Agriculture and Biology, Shanghai Jiao Tong University, Shanghai 200240, China; kimi1201@sjtu.edu.cn (Y.L.); donaghy-zhang@sjtu.edu.cn (D.Z.); jnding@sjtu.edu.cn (J.D.); khayat97@sjtu.edu.cn (K.H.); eileenyang1986@sjtu.edu.cn (X.Y.); xjzhan@sjtu.edu.cn (X.Z.); zhdsjtu@sjtu.edu.cn (D.Z.); ytlu@sjtu.edu.cn (Y.L.); 2 Key Laboratory of Urban Agriculture, Ministry of Agriculture and Rural Affairs, Shanghai 200240, China; 3 Bor S. Luh Food Safety Research Center, Shanghai Jiao Tong University, Shanghai 200240, China

**Keywords:** aptamer, cadmium, cyclic voltammetry, electrochemical aptasensor, Ti-modified Co_3_O_4_

## Abstract

The current work demonstrates an electrochemical aptasensor for sensitive determination of Cd^2+^ based on the Ti-modified Co_3_O_4_ nanoparticles. In this unlabeled system, Ti-modified Co_3_O_4_ nanoparticles act as current signal amplifiers modified on the screen-printed carbon electrode (SPCE) surface, while the derivative aptamer of Cd^2+^ works as a target recognizer. In addition, the sensing is based on the increase in electrochemical probe thionine current signal due to the binding of aptamer to Cd^2+^ via specific recognition. In the current study, key parameters, including aptamer concentration, pH, and incubation time were optimized, respectively, to ensure sensing performance. Cyclic voltammetry was used not only to characterize each preparation and optimization step, but also to profile the bindings of aptamer to Cd^2+^. Under optimal conditions, Cd^2+^ can be determined in a linear range of 0.20 to 15 ng/mL, with a detection limit of 0.49 ng/mL, significantly below the maximum concentration limit set by the U.S. Environmental Protection Agency. Based on comparative analysis and the results of recovery test with real samples, this simple, label-free but highly selective method has considerable potential and thus can be used as an in-situ environmental monitoring platform for Cd^2+^ testing.

## 1. Introduction

Cadmium (Cd^2+^) is a bioaccumulative, non-biodegradable and highly toxic heavy metal, which causes a variety of human health disorders [1,2,3]. To minimize the associated potential health risks, the maximum concentration in drinking water for Cd^2+^ is set at 5 ng/mL, as defined by the U.S. Environmental Protection Agency (EPA). Likewise, due to the high concern for determination and monitoring of Cd^2+^, a range of highly sensitive conventional techniques have been used, including electrothermal atomization atomic absorption spectroscopy (ET-AAS) [4,5], graphite furnace atomic absorption spectrometry (GFAAS) [6,7] and inductively coupled plasma mass spectroscopy (ICP-MS) [8,9,10]. All these conventional techniques are sensitive and accurate, but there are some limitations, such as laborious, time-consuming operation, and limited applicability for in-situ determination. In this regard, it is of paramount importance to develop a simple, convenient, and robust method for Cd^2+^ determination.

In recent decades, there has been a high level of attention for single-stranded oligonucleotide—aptamer since they were selected for the first time through the systematic evolution of ligands by exponential enrichment (SELEX) system [11,12]. Additionally, due to its high specificity, compatibility, environment-friendly properties, reliability and ease of synthesis and modification, aptamer opens up novel insights into both qualitative and quantitative determination of organic and inorganic targets such as pesticides [13,14], proteases [15], toxins [16,17], antibiotics [18], and heavy metals [19,20,21,22]. For instance, heavy metals including Pb^2+^, Hg^2+^, and Ag^+^ can be sensed and quantified through specific dimensional structures of aptamer/target complex as “G-quadruplex”, “T-Hg^2+^-T”, or “C-Ag^+^-C”, respectively [23,24,25]. Currently, two different aptamers of Cd^2+^ have been respectively selected by two independent groups [26,27]. Then, colorimetric [28] and fluorescent methods [26,29] were exploited to determine Cd^2+^. As for electrochemical methods, they are sensitive, and some portable devices are already commercial and available, including pocketSTAT2 (Ivium Technologies, Netherlands), PalmSens4 (Palmsens, Netherlands), μStat-i 400 (Metrohm, Switzerland) and CHI1200C (CH Instruments, China). In particular, electrochemical biosensors benefit a lot from screen-printed carbon electrode (SPCE), due to disposability, low cost, portability, customized fabrication and mass production [30,31]. They have considerable potential for environmental monitoring of targets in field. However, there is very limited data available in the literature regarding electrochemical biosensors based on SPCE and unlabeled aptamer for Cd^2+^ determination.

Generally, there are two essential indicators for a robust aptasensor, i.e., selectivity and sensitivity. The former one is basically ensured by high affinity of aptamer. As far as sensitivity is concerned, it is usual to have electrodes modify with functional nanomaterials. Their large surface area and unique nanostructure could boost electrons transfer rate, amplify electrical response, and enhance performance of biosensors [32,33]. Co_3_O_4_, a transition metal oxide with attractive electrocatalytic, electronic, and electrochemical properties, has been widely used in a wide range of applications [34]. In addition, most of the Co_3_O_4_-based nanocomposites developed to date have shown great potential in applications of Li-ion batteries [35], supercapacitor [36], electrocatalysts [37], and electrochemical sensors [38]. It has also been reported that their electron transfer and other electrochemical properties can be further promoted through modification with other transition metal oxides [35,39]. Anatase TiO_2_, on the other hand, is the most widely explored functional metal oxide [40], which has demonstrated importance in many electrochemical applications due to its large specific surface area and uniform nanopore distribution [41,42]. Its nanohybrids have also presented great advantages in electrical biosensors with high performance [43]. However, the synergetic effect of Ti-modified Co_3_O_4_ on electrochemical aptasensor was insufficiently investigated.

**Scheme 1:**
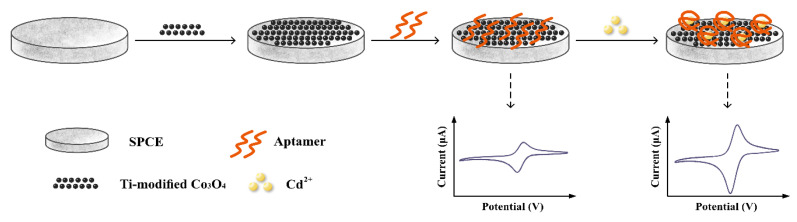
Schematic view of fabrication and principle of a label-free electrochemical aptasensor used for Cd^2+^ determination in the current study.

Based on the above, we have proposed a label-free electrochemical aptasensor for highly selective as well as sensitive determination of Cd^2+^, which consists of the derived aptamer and Ti-modified Co_3_O_4_ as a recognizer and a signal amplifier, respectively. The electrochemical properties and selectivity of the aptasensor are characterized under optimal conditions. Furthermore, aptamer-based methods and a recovery test with environmental samples will be compared and performed, respectively, to assess its practical feasibility at field level. Scheme 1 shows the architecture and signaling mechanism of the label-free electrochemical aptasensor.

## 2. Materials and Methods

### 2.1. Chemicals and Reagents

Ti-modified Co_3_O_4_ was synthesized as per the following steps. Briefly, Co(NO_3_)_2_·6H_2_O and Ti[O(CH_2_)_3_CH_3_]_4_ were dissolved in 80 mL of ultrapure water and 20 mL ethanol, respectively. Then, they were mixed with different molar ratios of Co:Ti (1:1 and 2:1) by stirring continuously for 3 h. Afterward, 10 mL of ammonia water was added dropwise and continuously stirred for 2 h. The resulting precipitates were finally rinsed three times with ultrapure water and separated using suction filtration followed by 10 h drying at 90 °C and 3 h calcination at 500 °C. Co_3_O_4_ was also prepared from Co(NO_3_)_2_·6H_2_O by following the same procedures with the omission of Ti[O(CH_2_)_3_CH_3_]_4_. According to the initial molar ratios of Co:Ti (1:1 and 2:1) mixed, samples obtained were marked as Co_1_Ti_1_ and Co_2_Ti_1_, respectively. [44]. The cadmium aptamer sequence originated from its parent [27] by subtly adding 5 nucleotides at 3′: 5′-GGA CTG TTG TGG TAT TAT TTT TGG TTG TGC AGT CC-3′, which was synthesized by Sangon Biotech Co. Standard solution of Cd^2+^ (1000 mg/L) was purchased from Merck Co., Inc. (Darmstadt, Germany). Thionine was purchased from Sinopharm Chemical Reagent Co. (Beijing, China). The detection buffer, a 0.1 M HAc-NaAc solution (containing 1.0 mM thionine), consisted of different volumes of thionine, sodium acetate and glacial acetic acid. Ammonia water was purchased from Shanghai Lingfeng Reagent Co. (Shanghai, China). All other reagents purchased from Sigma Aldrich (St. Louis, MO, USA) were used (in the current study) without further purification.

### 2.2. Apparatus

CHI1030A Electrochemical Workstation (CH Instruments, Shanghai, China) was used for electrochemical characterizations with an SPCE system (Jiangsu Rongbin Biotech Co., Nanjing, China), which contained (i) a carbon auxiliary electrode, (ii) Ag/AgCl reference electrode and (iii) a 2.0 mm diameter working electrode, respectively. All the ultrapure water utilized in this work was prepared by a Millipore-MilliQ system (Millipore Inc., Bedford, MA, USA). Scanning electron microscope (SEM, NOVA NanoSEM 230, Hillsboro, OR, USA), transmission electron microscope (TEM, TALOS F200X, Thermo Fisher Scientific, Waltham, MA, USA) and energy dispersive spectroscopy (EDS, TALOS F200X, Thermo Fisher Scientific, USA) were employed for characterization of as-prepared Co_3_O_4_ and Ti-modified Co_3_O_4_. X-ray diffraction patterns (XRD) were conducted on a D8 Advance Da Vinci Poly-functional X-Ray Diffractometer (Bruker Corporation, Billerica, MA, USA).

### 2.3. Aptasensor Fabrication Procedures

For drop-coating evenly on a bare electrode, the homodisperse of nanoparticles in water was required. Thus, only Co_2_Ti_1_ was adequate and then used for the aptasensor fabrication. 5.0 mg Co_2_Ti_1_ were added into 5.0 mL of ultrapure water and then ultrasonicated for 45 min. From this homogeneous dispersion mixture, 2.5 μL was drop-coated on the working electrode, followed by drying at room temperature for 1~2 h. Afterward, 2.5 μL of 2.0 μM Cd^2+^ aptamer was evenly introduced on the surface of the resulting electrode and dried for another 1~2 h to form a sensing interface, which was subsequently rinsed with ultrapure water to remove unbound ssDNA. Eventually, the aptasensor for Cd^2+^ determination was prepared after drying.

### 2.4. Electrochemical Measurements

The electrochemical measurements were performed by cyclic voltammetry (CV) in detection buffer (pH 5.5), which was swept from −0.65 to 0.00 V with a scan rate of 100 mV/s. Each measurement was repeated at least three times.

### 2.5. Determination of Cd^2+^ in Environmental Samples

Freshwater samples from a tributary of the Huangpu River and tap water at Minhang campus at Shanghai Jiao Tong University were collected to assess the reliability and operational capability of the proposed aptasensor through a recovery test. These samples were filtered through 0.22 µm membranes after adding various concentrations of Cd^2+^, followed by target determination via the proposed apparatus and procedure. Meanwhile, a standard approach, AAS, was employed as a contrast. Each measurement was repeated at least three times.

## 3. Results and Discussion

### 3.1. Characterization of Co_3_O_4_ and Co_2_Ti_1_

**Figure 1:**
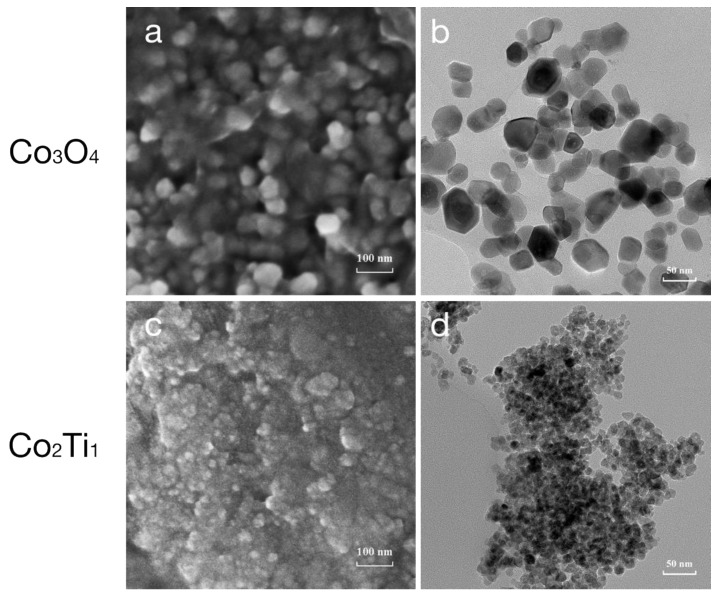
SEM and TEM images of Co_3_O_4_ (**a**,**b**) and Co_2_Ti_1_ (**c**,**d**).

**Figure 2:**
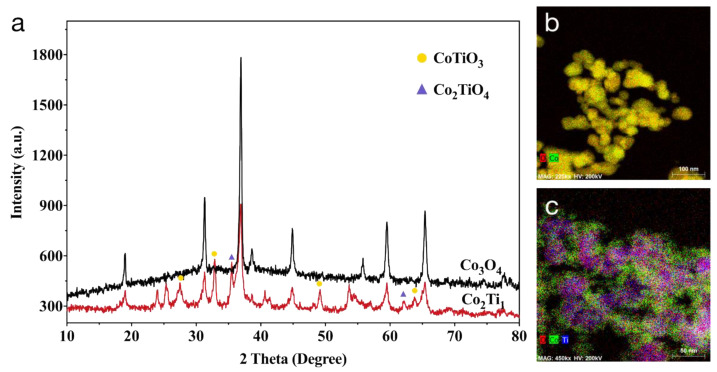
(**a**) XRD patterns and EDS elemental mapping images of (**b**) Co_3_O_4_ and (**c**) Co_2_Ti_1_.

The microscopic morphologies of Co_3_O_4_ and Co_2_Ti_1_ were respectively characterized by SEM, TEM, EDS, and XRD. As presented in Figure 1a,b, pure Co_3_O_4_ was up to 50 nm in size, while the size of Co_2_Ti_1_ varied from 10 to 20 nm after the incorporation of Ti (Figure 1c,d). As a consequence, the specific surface area enlarged remarkably, boosting both loading capacity and conductivity. The XRD patterns of Co_3_O_4_ and Co_2_Ti_1_ were also demonstrated in Figure 2a. It was easy to observe that the diffraction peaks of Co_2_Ti_1_ were weaker than those of pure Co_3_O_4_, indicating that the size of Co_3_O_4_ decreased obviously after the incorporation of Ti. Meanwhile, several weak peaks appeared in the diffraction pattern of Co_2_Ti_1_. Those peaks located at 27.4°, 32.7°, 48.9°, 63.6°, and 35.4°, 62.0° could be respectively assigned to CoTiO_3_ and Co_2_TiO_4_ [45,46], suggesting the formation of Co-O-Ti solid solutions. Finally, as shown in the images of EDS elemental mapping (Figure 2b,c), these three elements were distributed homogeneously. However, the content of Ti in Co_2_Ti_1_ was lower than that of Co and O (Figure 2c), which might be ascribed to the initial molar ratio of Co:Ti mixed in preparation.

### 3.2. Electrochemical Characterization of the Aptasensor

**Figure 3:**
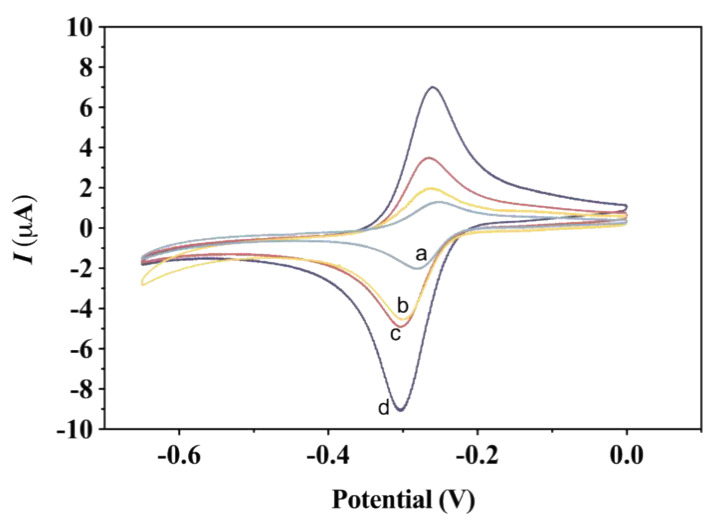
CV of electrode under each assembly procedure from −0.65 to 0.00 V (scan rate: 100 mV/s): (**a**) bare electrode, (**b**) electrode modified with Co_2_Ti_1_, (**c**) electrode modified with Co_2_Ti_1_/aptamer, (**d**) electrode modified with Co_2_Ti_1_/aptamer/Cd^2+^.

Cyclic voltammetry is a convenient and effective technique for the characterization of modified electrode. Therefore, the behaviors of the electrochemical probe after each assembly procedure were studied by this technique. As shown in line a of Figure 3, the thionine presented an insignificant response on bare electrode because of poor conductivity. However, after the Co_2_Ti_1_ modification, the peak currents increased significantly (line b of Figure 3), due to its impact on specific surface area expansion and electron-transfer promotion. As a result, the sensitivity changed positively together with the detection limit of the aptasensor. The peak current increased slightly since the introduction of the aptamer (line c of Figure 3), indicating that it was successfully immobilized on working electrode. The possible reason for the current difference may be ascribed to the weak electrostatic attraction between the positively charged thionine and the negatively charged phosphate groups of the DNA backbone [47,48], resulting in its adsorption along with adhesion of aptamer to electrode, thus gently facilitating electron-transfer. Finally, after incubating the aptasensor with Cd^2+^ (line d of Figure 3), a significant peak current was observed, indicating the formation of Cd^2+^-aptamer complex through specific recognition. In short, a weak current signal could only be observed in absence of Cd^2+^, while a strong electrochemical signal could be detected in the presence of Cd^2+^ due to the Cd^2+^-aptamer complex, thereby confirming that the aptasensor was successfully prepared.

### 3.3. Optimization of Experimental Conditions

In addition to a profiling technique, CV also served as a sensitive electrochemical analysis method. In this regard, the experimental conditions including aptamer concentration, pH value and incubation time were optimized to achieve optimal performance. Respective data about the optimization results are presented in the Supplementary Material (Figure S1). In brief, the optimal conditions obtained were: (a) aptamer concentration: 2.0 μM; (b) pH value: 5.5; (c) incubation time: 40 min.

### 3.4. Analytical Performance

**Figure 4:**
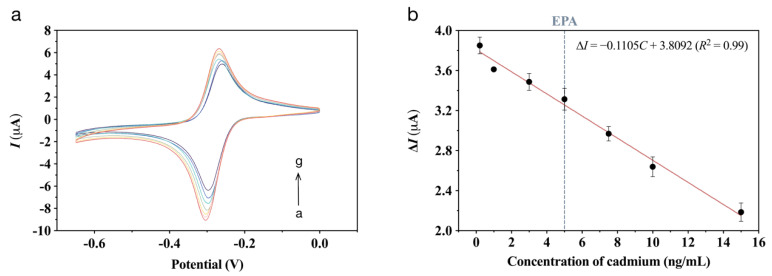
(**a**) CV responses to aptasensor upon various concentrations of Cd^2+^ from a to g: (a) 0.2, (b) 1.0, (c) 3.0, (d) 5.0, (e) 7.5, (f) 10.0, (g) 15.0 ng/mL in detection buffer (pH = 5.5); (**b**) Calibration plots of Δ*I* versus concentration of Cd^2+^.

The analytical performance of the aptasensor was evaluated under the aforementioned optimal experimental conditions, where a series of Cd^2+^ determinations were performed to estimate the sensitivity. The Δ*I* value (variation of peak current signal before and after the introduction of Cd^2+^ to electrode) decreased as increasing concentration of Cd^2+^, which also presented a good linearity from 0.20 to 15 ng/mL (Figure 4b). The linear regression equations determined were:

∆*I* = −0.1105*C* + 3.8092 (*R*^2^ = 0.99) (1)

(*C*: the concentration of Cd^2+^; *R*^2^: correlation coefficient). 

The limit of detection (LOD) was estimated as 0.49 ng/mL (3σ/slope), which is significantly lower than the maximum concentration limit set by EPA. Moreover, on the basis of the comparative analysis of aptamer-based approaches (Table 1), this aptasensor is more sensitive than most of the others. It is noteworthy that the LOD of this method is about 21-fold lower than that of another electrochemical aptasensor, demonstrating its high sensitivity. Although a fluorescent aptasensor has a significantly lower LOD, it is unable to quantify Cd^2+^ below 100 ng/mL, to which the current electrochemical aptasensor is well complementary.

### 3.5. Selectivity and Reproducibility

**Figure 5:**
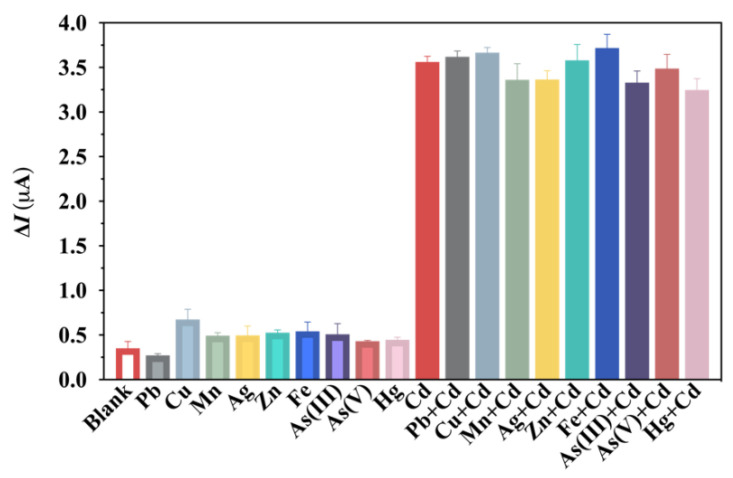
Selectivity of the aptasensor for Cd^2+^ determination. The concentrations of interfering ions were 50.0 ng/mL, and Cd^2+^ concentration was 2.0 ng/mL.

The selectivity performance of the current method was determined by recording current signal variations (Δ*I*) of potential interfering ions in the absence and presence of Cd^2+^. The concentrations of interferences (e.g., Pb^2+^, Cu^2+^, Mn^2+^, Ag^+^, Zn^2+^, Fe^2+^, As(III), As(V) and Hg^2+^) were 50.0 ng/mL, while Cd^2+^ concentration was 2.0 ng/mL. As shown in Figure 5, the Δ*I* values of sensing systems containing a higher concentration of interfering ions were significantly less than that added with Cd^2+^. Nevertheless, those recovered sharply after the introduction of Cd^2+^ to the sensing system containing interfering ions. These results enabled confirmation that the interfering ions could negligibly affect Cd^2+^ determination, even if their concentrations were 25 times higher than that of Cd^2+^, which confirmed excellent selectivity of the proposed aptasensor for Cd^2+^ determination.

Furthermore, according to possible secondary structures predicted preliminarily by UNAfold (https://sg.idtdna.com/UNAfold/) (Figure S2), the subtle addition with 5 nucleotides at 3′ of this aptamer successfully induced its preferential configuration of a stem-loop structure (Figure S2a) from a random coil sequence (Figure S2b) via conformational switching. Meanwhile, Cd^2+^ was incorporated and buried with the binding pocket of this structure, underlying its high sensitivity and selectivity. As structures of “G-quadruplex” and “T-Hg^2+^-T” have been sufficiently studied, we are able to develop various aptasensors for Pb^2+^ and Hg^2+^ detection. Unfortunately, the mechanism of how aptamer bind Cd^2+^ is not yet fully understood. To elucidate the fundamental and further development of Cd^2+^ aptasensor, a lot of effort is still needed.

Under the same conditions, reproducibility was also examined at a Cd^2+^ concentration of 2.0 ng/mL using 10 identically prepared SPCEs. The obtained results of the relative standard deviation (RSD) of 4.37% indicated that the reproducibility of this aptasensor was quite acceptable.

### 3.6. Application in Environmental Samples

As demonstrated in the selectivity investigation previously, the proposed aptasensor presented strong anti-interference capability against heavy metal ions. However, environmental samples were more complicated, in which various species of interferences might exist, e.g., microorganisms, biometabolites, and antibiotics. To assess the applicability of a new method to those complicated samples, a recovery test was widely adopted and accepted. Therefore, the recovery test with real samples was performed to analyze the validity of the current method, while AAS was employed as a contrast. Table 2 exhibited the mean recoveries of the Cd^2+^ and RSD values in two Cd^2+^ spiked samples obtained with the proposed aptasensor and AAS. The amounts of Cd^2+^ added were set according to the EPA limit (5 ng/mL). As shown in Table 2, the recoveries of this method ranged from 98.71% to 109.95% with RSD values of 5.75% to 8.24%. In consideration of its simplicity, convenience, analytical performance and application scenarios, these comparable recoveries with AAS were well acceptable. All of the above results substantiated its practicability and promising future for in-situ monitoring of Cd^2+^ in field.

## 4. Conclusions

In conclusion, the effectiveness of a robust electrochemical aptasensor based on the derivative aptamer and Ti-modified Co_3_O_4_ was summarized, which was designed for highly sensitive and selective determination of Cd^2+^ in environmental samples. The basic principle for Cd^2+^ determination underlies an increase in the current signal of electrochemical probe thionine due to the formation of Cd^2+^-aptamer complex through specific recognition. The detection limit obtained (0.49 ng/mL) is significantly lower than the limit set by the U.S. EPA, indicating its possible use as an environmental monitoring platform for Cd^2+^. Although the system possesses merits as aforementioned, there are still some limitations existing. For example, only one target can be detected in a measurement. Extra and tedious pretreatment is also needed for complex samples. Its availability can be improved if either simultaneous measurements of multiple heavy metal ions or integration with auto-pretreatment into a portable device is realized. Furthermore, the proposed aptasensor was based on an SPCE system, which meant each channel was one-off. The capability of being regenerated should be taken into serious consideration along with aptasensor development. Therefore, our future work will be carried out to address the above issues.

## Figures and Tables

**Table 1:** Comparison of different aptamer-based approaches for Cd^2+^ determination.

Approaches	Linear Range (ng/mL)	LOD (ng/mL)	Reference
fluorometry	0.00~112.41	4.50	[26]
	100.00~10,000.00	0.04	[29]
colorimetry	1.00~400.00	1.00	[28]
	1.12~44.96	0.52	[27]
electrochemistry	28.10~112.41	10.34	[49]
	0.20~15.00	0.49	This work

**Table 2:** Determination of Cd^2+^ in environmental samples (n = 3).

Sample	Background (ng/mL)	Spiked (ng/mL)	Proposed Electrochemical Aptasensor			AAS		
			Found (ng/mL)	Recovery (%)	RSD (%)	Found (ng/mL)	Recovery (%)	RSD (%)
River water	0.02	3.00	3.18	105.24	8.17	3.09	102.17	0.66
		6.00	6.12	101.73	5.75	5.99	99.48	0.46
		12.00	13.03	108.43	6.11	12.75	106.03	1.50
Tap water	0.07	3.00	3.03	98.71	8.24	3.20	104.22	0.18
		6.00	6.22	102.43	7.43	6.29	103.69	0.82
		12.00	13.27	109.95	7.64	12.82	106.19	2.48

## Supplementary Materials

The following are available online at https://www.mdpi.com/2079-6374/10/12/195/s1, Figure S1: Optimization of the experimental parameters: (a) aptamer concentration, (b) pH of buffer, (c) incubation time on aptasensor with Cd^2+^ by CV in detection buffer (0.1 M HAc-NaAc solution containing 1.0 mM thionine), Figure S2: Possible secondary structures of aptamer predicted by UNAfold (https://sg.idtdna.com/UNAfold/): (a) most stable stem-loop structure form and (b) most stable random coil sequence form.
