# Supplementary material for: Label-Free and Sensitive Determination of Cadmium Ions Using a Ti-Modified Co3O4-Based Electrochemical Aptasensor
Source: Biosensors (Basel). 2020 Nov 30;10(12):195. doi: 10.3390/bios10120195 (PMC7761109; doi:10.3390/bios10120195)
Supplement: Supplementary file 1 [file biosensors-10-00195-s001.pdf]

biosensors

MDPI

Electronic Supplementary Material

Label-Free and Sensitive Determination of Cadmium Ions Using a Ti-Modified Co_3O_4-Based Electrochemical Aptasensor

Yang Liu [1,2,3], Dongwei Zhang [1,2,3], Jina Ding [1,2,3], Kashif Hayat [1,2,3], Xijia Yang [1,2,3], Xuejia Zhan [1,2,3], Dan Zhang [1,2,3], Yitong Lu [1,2,3] and Pei Zhou [1,2,3,*]

[1] School of Agriculture and Biology, Shanghai Jiao Tong University, Shanghai 200240, China;
[2] Key Laboratory of Urban Agriculture, Ministry of Agriculture and Rural Affairs, Shanghai 200240, China;
[3] Bor S. Luh Food Safety Research Center, Shanghai Jiao Tong University, Shanghai 200240, China; kimi1201@sjtu.edu.cn (Y.L.); donaghy-zhang@sjtu.edu.cn (D.Z.); jnding@sjtu.edu.cn (J.D.); khayat97@sjtu.edu.cn (K.H.); eileenyang1986@sjtu.edu.cn (X.Y.); xjzhan@sjtu.edu.cn (X.Z.); zhdsjtu@sjtu.edu.cn (D.Z.); ytlu@sjtu.edu.cn (Y.L.)
* Correspondence: zhoupei@sjtu.edu.cn; Tel.: +86-021-34205762

Received: 30 October 2020; Accepted: 26 November 2020; Published: 30 November 2020

Optimization of Experimental Conditions

CV peak currents and ΔI were used for the optimization of experimental conditions, where ΔI was the change of peak response before and after introduction of Cd^{2+}. At least four replicates were performed for each treatment.

At first, the effect of aptamer concentration on signal response was investigated. The aptamer with various concentrations was incubated with 2.0 ng/mL of Cd^{2+} for 60 mins. As shown in Figure S1a, ΔI rose markedly with increasing aptamer concentration, and tended to a steady value when it was 2.0 μM, indicating all the aptamer had bound with Cd^{2+}. Therefore, 2.0 μM was chosen as the optimal aptamer concentration.

As a vital factor influencing sensing system, the effect of pH value was also optimized. Figure S2b reveals that the maximum reduction peak current was obtained at pH = 5.5, hence pH value of 5.5 was selected for Cd^{2+} determination.

Finally, the effect of incubation time on the performance of the aptasensor was studied. As depicted in Figure S3c, ΔI almost kept constant after 40 minutes' incubation, suggesting that 40 mins was suitable for thoroughly capture of target on the aptasensor surface. Thus, 40 mins was adopted in the following trials.

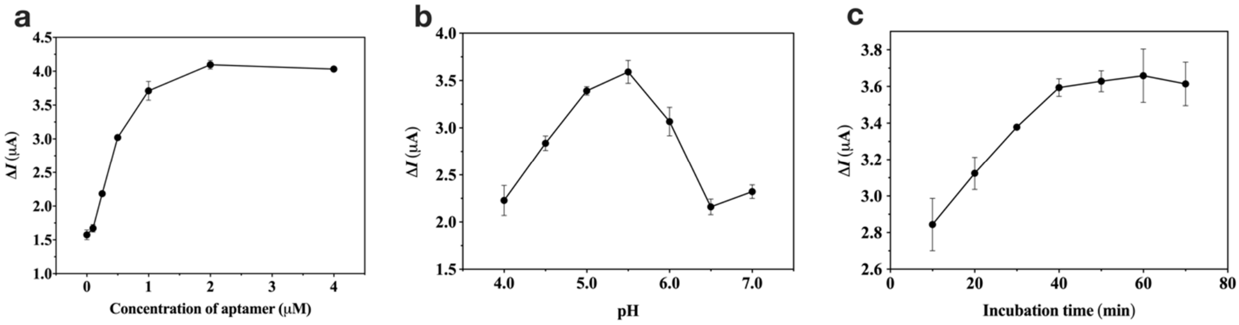

Figure S1. Optimization of the experimental parameters: **(a)** aptamer concentration, **(b)** pH of buffer, **(c)** incubation time on aptasensor with Cd^{2+} by CV in detection buffer (0.1 M HAc-NaAc solution containing 1.0 mM thionine).

Possible Secondary Structures of Aptamer Predicted by UNAfold

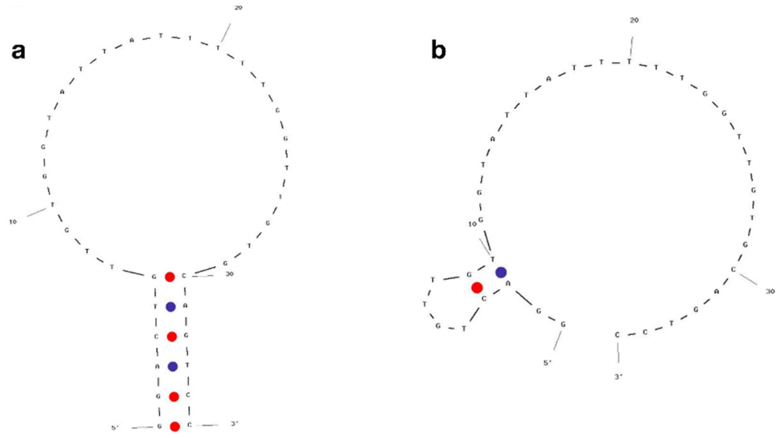

Figure S2. Possible secondary structures of aptamer predicted by UNAfold (https://sg.idtdna.com/UNAfold/): **(a)** most stable stem-loop structure form and **(b)** most stable random coil sequence form.

© 2020 by the authors. Licensee MDPI, Basel, Switzerland. This article is an open access article distributed under the terms and conditions of the Creative Commons Attribution (CC BY) license (http://creativecommons.org/licenses/by/4.0/).
